# Proof-of-concept randomised controlled trial of data-driven hearing rehabilitation versus standard care in older adults with hearing loss: the healthy hearing for healthy ageing protocol

**DOI:** 10.1136/bmjopen-2026-122681

**Published:** 2026-07-21

**Authors:** Laura Ihalainen, Mariagnese Barbera, Timo Törmäkangas, Petteri Hyvärinen, Tytti Willberg, Pia Linder, Alina Solomon, Aarno Dietz

**Affiliations:** 1 Department of Otorhinolaryngology, Kuopio University Hospital, Kuopio, Finland; 2 Institute of Clinical Medicine, University of Eastern Finland, Kuopio, Finland; 3 The Ageing Epidemiology Research Unit, School of Public Health, Imperial College London, Charing Cross Hospital, London, UK; 4 Gerontology Research Center, Faculty of Sport and Health Sciences, Jyvaskylan Yliopisto, Jyvaskyla, Finland; 5 Department of Otorhinolaryngology, Turku University Central Hospital, Turku, Southwest Finland, Finland; 6 Division of Clinical Geriatrics, Center for Alzheimer Research, Department of Neurobiology, Care Sciences and Society, Karolinska Institutet, Stockholm, Sweden

**Keywords:** Clinical Trial, Hearing, Cognition, REHABILITATION MEDICINE

## Abstract

**Introduction:**

Hearing aids (HAs) can alleviate hearing loss; however, HA rehabilitation is frequently hampered by delayed diagnosis, suboptimal fitting and lack of systematic follow-up. Although the association between hearing loss and cognitive decline has been identified, evidence from randomised controlled trials remains limited. Addressing these gaps is essential for reducing hearing loss-related experiences and evaluating whether effective HA use could reduce the risk of cognitive decline.

**Methods and analysis:**

The healthy hearing for healthy ageing study is a proof-of-concept, single-site, two-arm parallel-group 12-month randomised controlled trial with a 12-month extended follow-up. Up to two hundred participants with hearing loss and without cognitive decline referred for an initial HA rehabilitation are recruited and randomised 1:1 to either data-driven hearing rehabilitation or standard care. The primary outcome is the change in two speech perception in noise tests validated for the Finnish language: the Finnish matrix sentence test and the digits-in-noise test. The secondary outcomes are patient-reported outcomes (eg, Hearing in Real-Life Environment and Speech, Spatial and Quality questionnaires), quality of life (eg, 15D-questionnaire), cognitive (eg, Consortium to Establish a Registry for Alzheimer’s Disease test) and psychosocial measures. Exploratory outcomes include event-related responses, cortical auditory evoked potentials, structural brain imaging and vision-related measures.

**Ethics and dissemination:**

Ethical approval has been obtained from the Regional Medical Research Ethics Committee of the well-being Services County of North Savo (approval no. 697/2023). Findings from this study will be disseminated through peer-reviewed publications, conference presentations and relevant clinical and patient communities.

**Trial registration number:**

NCT06495268

**STRENGTHS AND LIMITATIONS OF THIS STUDY**

- Embedding the trial in routine clinical practice with recruitment through clinical referrals enhances real-world relevance and reduces selection bias.
- The randomised controlled design, large sample size (N up to 200) and extended duration strengthen the study in a previously underexplored area.
- The usual-care control arm supports real-world applicability, with variability managed through systematic tracking of key rehabilitation variables.
- Double-masking is implemented as far as feasible: all participants receive identical hearing aids, assessors are masked and participants are not explicitly informed of group allocation.
- Follow-up may be limited to capture substantial cognitive decline or incident dementia, although extended follow-up is planned.

## Introduction

 Hearing loss is a major public health concern affecting 20% of the global population (6% with moderate to complete, ie, disabling hearing loss).^1^ Hearing loss is among the leading causes of years lived with disability in older adults.^1^ Due to the ageing of the population, it is estimated that over 900 million people will have significant hearing loss by the year 2050.^2^ The Lancet Commission on dementia prevention, intervention and care identifies hearing loss as one of the most relevant, potentially modifiable risk factors for cognitive decline and dementia. The Commission estimated that eliminating hearing loss in mid-life might reduce the risk of dementia by 7%.^3^ Even though a causal relation between hearing loss and cognitive decline is yet to be established, hearing rehabilitation may be an effective intervention for reducing risk factors such as social isolation, anxiety and depression, which have been linked to hearing loss as well as cognitive decline.^4^

A recently published multicentre randomised trial (The Ageing and Cognitive Health Evaluation in Elders Study) demonstrated that hearing intervention reduced cognitive decline by 48% over 3 years in older adult participants with other risk factors for dementia.^5^ The underlying mechanisms by which hearing interventions may slow down cognitive decline remain largely unknown. Therefore, to substantiate the role of hearing interventions in reducing the risk of cognitive decline, clinical studies are needed to comprehensively explore various outcome measures and identify those that are most relevant to promote healthy ageing.

Findings on the factors determining the success of hearing aid (HA) interventions vary widely, and there is no consensus on the most relevant outcome measures for evaluating these interventions.^6 7^ This lack of agreement contributes to the infrequent validation of hearing rehabilitation outcomes in clinical settings. Consequently, there is a surprising paucity of clinical data on real-world HA use and its benefits, which hinders evidence-based decision-making.^6^ Additionally, the absence of systematic follow-up may help explain the low levels of HA adoption and compliance.

Currently, the success of hearing rehabilitation is most often evaluated using patient-reported outcome measures (PROMs). Despite being low cost, PROMs are rarely implemented in hearing rehabilitation processes for older adults in clinics. While PROMs provide valuable insights into a patient’s subjective experiences and perceptions, they are insufficient and unreliable for evaluating audiological outcomes, as the results do not necessarily align with objective measurements.^8^ Older adults, in particular, have been shown to underestimate the degree of their hearing loss in PROMs.^9^ Additionally, many hearing-related PROMs have not been adequately validated, further decreasing their reliability.

Performance-based measures, such as speech intelligibility in noise (SPIN), can be particularly effective in capturing the everyday challenges posed by hearing loss. SPIN evaluates an individual’s ability to understand speech in noisy or reverberant environments and is currently the most appropriate measure of the real-world impact of hearing loss. Therefore, systematically measuring pre- and post-intervention SPIN could provide valuable data about the relevant factors for hearing rehabilitation.

Electrophysiological measurements of auditory pathways would represent another promising approach for the objective assessment of hearing loss and HA effects. Clinical studies have demonstrated that regular HA use can modify the auditory system, often referred to as acclimatisation to HA use. Acclimatisation is a process in which HA users not only become gradually accustomed to amplification but also gradually develop better hearing performance. However, the potential extent of adaptation of a given individual’s auditory system remains largely unknown.^10 11^ It is also unclear whether HAs can induce beneficial plastic changes in perceptual functions relevant to SPIN. The possible neural mechanisms triggered by HA use are still unknown, and if neural changes occur, the physiological mechanisms underlying these changes are yet to be determined.^12^

Dual sensory impairment (DSI) refers to the presence of both hearing and vision loss, with a prevalence between 10% and 20% in older adults (> 70 years).^13^ Previously, hearing and vision loss have been independently linked to a higher risk of cognitive decline; however, the impact of DSI on this risk remains inconclusive.^14^ It has been demonstrated that the concerns in DSI are considerably greater than the effects of vision or hearing loss alone, since individuals with dual sensory differences may experience greater activity limitations and participation restrictions than those with hearing or vision loss alone.^15^ Currently, there are no best-practice guidelines for the rehabilitation of individuals with DSI.

The healthy hearing for healthy ageing (HAHA) study is a randomised controlled trial (RCT) investigating whether considering hearing loss as a broader degenerative condition can improve both hearing and cognitive outcomes. This trial compares a data-driven hearing rehabilitation (DDHR) programme with standard care in older adults with mild to moderately severe hearing loss and without cognitive decline. DDHR is an evidence-based rehabilitation programme developed for this study in accordance with current best practices in hearing rehabilitation. Standard care corresponds to the current model of hearing rehabilitation in Finland, as described in more detail in the Methods section. The primary outcome measure is the improvement in SPIN following HA rehabilitation, serving as a key indicator for hearing function. In addition, this trial aims to address the broader neural and cognitive mechanisms, which is why we included secondary outcome measures assessing auditory processing and brain function, including structural MRI, as well as cortical auditory evoked potentials (CAEPs). These measures are intended to explore whether more effective hearing rehabilitation, reflected in improved real-world listening function, is associated with measurable neural or cognitive changes. While the DDHR programme evaluated in this trial is intended to optimise functional hearing outcomes through a more structured rehabilitation process, its implementation may require additional clinical time and resources that are not routinely available in everyday audiology practice. Consequently, an important secondary objective of this proof-of-concept study is also to evaluate the feasibility of the DDHR programme and identify strategies to support its implementation in routine clinical care. Eventually, the HAHA trial aims to establish a personalised, data-driven approach to hearing rehabilitation that could contribute to healthy ageing in older adults with hearing loss.

## Methods and analysis

### Study design

**Figure 1:**
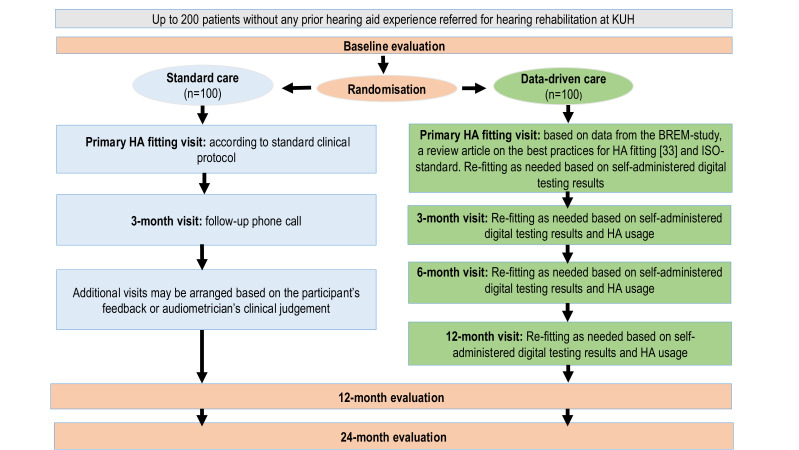
Study diagram. BREM, benefits of real-ear measurements; HA, hearing aid; ISO, International Organization for Standardization; KUH, Kuopio University Hospital.

HAHA is a proof-of-concept, single-site, two-arm parallel group, 12-month RCT with a 12-month extended follow-up (figure 1). Eligible participants are randomly allocated 1:1 to either the DDHR programme (active group) or the standard care (control group). In this study, ‘data-driven hearing rehabilitation’ refers to a structured, clinician-led fitting approach in which established verification procedures are used to ensure accurate amplification, combined with the systematic use of objective outcome measures (eg, SPIN performance, HA usage data and PROMs) to guide rehabilitation decisions according to predefined criteria. This approach is clinician-implemented and does not involve algorithmic or artificial intelligence-based decision-making. Standard care corresponds to the routine hearing rehabilitation programme currently provided at the Kuopio University Hospital (KUH) ENT clinic. HAs will be fitted using the manufacturers’ proprietary fitting algorithms, and real-ear measurements (REM) will be performed only in complex cases, based on the clinical judgement of the audiometrician. The main difference is that routine standard care does not typically include systematic assessment of rehabilitation outcomes, which may contribute to suboptimal outcomes and reduced adherence to HA use. To ensure a balanced distribution between the groups, randomisation is performed using a computer-generated list in blocks of four and with sealed envelopes. The list and the envelopes are prepared by a biostatistician and used by a study nurse to randomise the participants. Although double-masking is not possible, outcome assessors will be masked to the group allocation. Participants will not be actively informed of their group allocation; however, complete masking may not be feasible based on differences between the HA fitting protocols. The HAHA trial is completely embedded in clinical practice, and all trial participants will receive the same HAs as in routine care. Due to its pragmatic approach, the HAHA trial will follow the standard care wait-time (3–6 months) for the primary HA fitting visit, which marks the beginning of the intervention. Analyses will follow CONSORT guidelines^16^ and will include the intention-to-treat population, that is, all randomised participants, except for those who withdraw their consent (including for use of data). The Standard Protocol Items: Recommendations for Interventional Trials checklist^17 18^ is also included in the present protocol paper (online supplemental material S1).

### Participants

Up to 200 older adults (65–84 years of age) with mild to moderately severe sensorineural hearing loss^1^ are recruited from October 2024 onwards at the KUH among patients referred to the ENT clinic for HA rehabilitation of sensorineural hearing loss. Patients with conductive or asymmetrical hearing loss, HA contraindications, diagnosed cognitive impairment or cognitive concern under evaluation or any health condition affecting their ability to participate in the study are excluded. The eligibility criteria for the trial are presented in table 1. Patients are first screened as part of their routine hearing loss assessment appointment by an ENT specialist, and eligible patients are invited to participate in the study. A study nurse will obtain informed consent from the participants during the same appointment (online supplemental material S2). The recruitment for this study started on 1 October 2024, and the anticipated primary completion date is estimated to be 29 February 2028.

**Table 1:** Eligibility criteria

Inclusion criteria	Exclusion criteria
Age 65–84 years	Conductive hearing impairment (air-bone gap>20 dB HL in two consecutive frequencies)
Mild to moderately severe sensorineural hearing loss, pure-tone average (0.5–4 kHz) between 20 and 64 dB*	Difference between the hearing levels of the ears is >15 dB HL in three consecutive frequencies
Community-dwelling, that is, living at home/not living in a care home or nursing home	HA contraindication
Proficiency in Finnish language	Cognitive status: Previously diagnosed dementia or current use of cholinesterase inhibitors and/or memantine. If there is a current ongoing diagnostic process for suspected dementia, the decision on eligibility will be made by an experienced study physician based on medical records and clinical judgement.
First-time HA user	Any health conditions severely impairing vision (no predefined visual acuity threshold), mobility, communication or ability to participate in study visits and complete study assessments, as judged by the study nurse and/or physician.

* As per the Global Burden of Disease Expert Group on hearing loss criteria.

dB HL, decibel hearing level; HA, hearing aid.

### Patient and public involvement

Patients and/or the public were not involved in the design, conduct, reporting or dissemination plans of this research. Patients will be recruited only as study participants.

### Data collection

Eligible participants (table 1) are undergoing baseline assessments prior to randomisation. The same outcome assessments will be repeated at 12-month and 24-month follow-up visits after the beginning of the intervention, which is defined by the date of the first HA fitting appointment. Table 2 and online supplemental material S3 summarise the time schedule of the HAHA assessments. All outcome assessments are performed by study nurses specifically trained in the HAHA study procedures, who are masked to participants’ group allocation.

**Table 2:** Schedule of enrolment, interventions and assessments of the HAHA trial

	Study period						
	Pre-enrolment	Enrolment baseline assessment and allocation*	Post-allocation				Close-out
TIMEPOINT**	Screening	Baseline	Primary fitting visit (t_0_)	3 M	6 M	12 M	24 M
ENROLMENT:							
Eligibility screening†	X						
Informed consent		X					
Allocation		X					
INTERVENTIONS:							
DDHR intervention			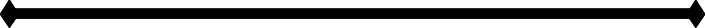				
Standard care			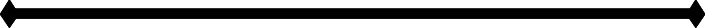				
Primary HA fitting (all participants)			X				
Re-fitting DDHR				X‡	X‡	X‡	X‡
Standard care follow-up phone call				X			
Standard care possible additional visits§				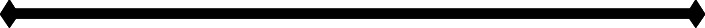			
ASSESSMENTS:							
Screening and baseline variables¶	X	X					
Outcomes variables**		X				X	X
REAR measures††			X				

* Enrolment will take place after the screening assessment that is conducted as part of the standard ENT clinical and diagnostic work-up; to prevent allocation affecting the baseline assessments, the primary outcome assessments will be conducted before allocation. The intervention will start as soon as possible after allocation, based on the timeline for HA fitting as part of the standard clinical management of the patients.

† The screening assessment is conducted prior to enrolment as part of the standard ENT clinical and diagnostic work-up.

‡ As needed based on participant feedback, the DIN test results and/or clinical judgement.

§ As needed based on participant request.

¶ For the full list of assessments, see the ‘Eligibility’ and ‘General Covariates’ sections of the Supplementary material S3.

** For the full list of assessments see ‘General Covariates’; ‘Medical History’; ‘Anthropometrics’; ‘Primary Outcomes’; ‘Secondary Outcomes’; ‘Cognitive Measures’; ‘Quality of life and other psycho-social measures’; ‘Exploratory/Optional Assessments’; ‘Vision-related measures’ and ‘Blood samples’ sections of the Supplementary material 3.

†† DDHR group.

DDHR, data-driven hearing rehabilitation; DIN, digits-in-noise; ENT, ear, nose and throat; HA, hearing aid; 3 M, 6 M, 12 M and 24 M, 3, 6, 12 and 24 months from primary fitting visit (t_0_); REAR, real-ear aided response.

#### Primary outcomes

Change in SPIN will be assessed with two tests/outcome measures: the Finnish matrix sentence test (FMST)^19^ and digits-in-noise (DIN) test.^20 21^

1. FMST is a functional audiological test validated for the Finnish language.^19^ In the test, participants listen to 5-word sentences chosen from a standardised and optimised word matrix, which allows for 105 different sentences of equal intelligibility. Each test list contains 20 randomly chosen sentences. The sentences are presented to the participants under stationary background noise (65 dB SPL) generated from the speech material of the FMST. The test is presented in the S0N0 condition, which means that the signal and background noise are presented through one loudspeaker at a 0° azimuth. Three test lists will be presented to the participants. The participants repeat all the words they can identify, and they are allowed also to guess words that they could not hear clearly. The result of the FMST is the signal-to-noise ratio (SNR) at which the participant correctly identifies 50% of the presented word items; this is called the speech reception threshold (SRT50).
2. DIN is a functional hearing test^20^ validated for the Finnish language.^21^ The word material of the test consists of digit triplets presented under stationary background noise (65 dB SPL). The result of DIN is the SNR at which the participant correctly identifies 50% of the presented triplets (SRT50). The DIN will be performed as a closed-set test, which means that the participant types their responses via a touch screen. Participants will be tested during the baseline visit without HAs and during the following visits (12 and 24 months) with and without HAs in both ears simultaneously.

#### Secondary outcomes

Changes in the following secondary endpoints will be included: Hearing in Real-Life Environment (HERE)^22^ and Speech, Spatial and Qualities of Hearing Scale (SSQ)^23^; HA usage, self-report and HAs log-data; listening effort questionnaire^24^; response time of the DIN test and tinnitus handicap inventory (THI)^25^; Consortium to Establish a Registry for Alzheimer’s Disease -test’s (CERAD-nb) global and domain scores^26^; Clinical Dementia Rating Sum of Boxes^27^; health-related quality of life (15D and EQ5-D-5L questionnaires^28 29^); self-reported depression symptoms and Beck Depression Inventory^30^ (table 2, online supplemental materials S3 and S4).

#### Exploratory outcomes

A range of exploratory analyses will investigate potential intervention effects on active lifestyle (change in lifestyle factors related to cognitive decline, eg, physical, cognitive and social activities) as well as accessible and non-invasive biomarkers as possible predictors of hearing rehabilitation benefits. These biomarkers include electroencephalogram (EEG)-derived CAEPs, in which the analysis focuses on the amplitude, latency and duration of the sustained potential of the speech-evoked response and brain structural MRIs probing the effects of hearing impairment and rehabilitation on volumetric measures. The full list of exploratory outcome measures is presented in online supplemental material S3.

#### Other data collected

Other relevant data will be collected, including demographics, medical history, anthropometrics, subjective memory questionnaire,^31^ Basic Nordic Sleeping Questionnaire^32^ and other lifestyle-related risk factors for cognitive decline (eg, diet, smoking and alcohol consumption). Audiograms will be obtained through pure-tone audiometry using an Aurical Aud audiometer (Natus Medical Incorporated, Middleton, WI, US) with TDH39 audiometric headphones (TTM Technologies Inc., St. Louis, MO, US) in a soundproof booth, following international standards (ISO 8253–1:2010). Air conduction (AC: 250–8000 Hz) and bone conduction (BC: 250–4000 Hz) measurements are performed by experienced audiometricians. The pure-tone average is calculated for AC (mean of 500, 1000, 2000 and 4000 Hz). Vision-related measures (ie, best-corrected visual acuity, slit-lamp examination, retinal imaging, eye geometrics and biometrics values) are collected to investigate the impact of dual (hearing and vision) sensory impairment on cognition. At baseline and 24 months, non-fasting venous blood samples will be collected from all participants and stored for future measurements of biomarkers for diseases related to cognitive decline (eg, genetics related to amyloid, τ, neurodegeneration or other future relevant biomarkers).

#### Safety reporting

HAs are safe and well tolerated, with no known serious health risks. Pre-specified adverse events (eg, ear canal infection, skin conditions) and serious adverse events will be monitored throughout the trial by an independent Data and Safety Monitoring Board.

### Study intervention

Participants in the DDHR intervention group will receive data-driven and regularly controlled HA rehabilitation that is not part of the standard care protocol. Prescription HAs with custom earmoulds will be fitted by using REM in accordance with the recommendations of the latest ISO standard (21388:2020) and a recent review article on the best practices for HA fitting.^33^ In addition, preliminary unpublished findings from the benefits of REM (BREM) study (ClinicalTrials.gov identifier: NCT05621798) suggest that REM-based fitting may result in improved SPIN performance compared with conventional fitting methods. The fittings are conducted by audiometricians specifically trained in the DDHR programme procedures. The gain of sound amplification is set to 100%. After the fitting of the HAs, participants’ auditory-related outcomes will be assessed at the 3-, 6- and 12-month follow-up visits using the DIN test. If needed, re-fitting will be performed based on the following criteria derived from the preliminary data of the BREM study:

1. The absolute improvement in the DIN<1.5 dB (SNR), OR
2. The absolute result of the DIN test>than −8.5 dB (SNR), OR
3. The participant is unsatisfied with the HA fitting.

If re-fitting is needed, the log-data of the HAs will be verified, and the following procedures will be performed based on the audiometrician’s clinical experience:

1. Overall usage <6 hour/day: The reason for low usage is probed, and the participant is encouraged to use the HA more. If the amplification of the HA is reported to be too loud, the sound amplification gain can be lowered to 90%. Fine-tuning of specific frequencies is also allowed based on the participants’ feedback.
2. Overall usage ≥6 hour/day: The participant’s satisfaction with ongoing HA rehabilitation is inquired. If the participant is satisfied, no further adjustments are needed. If the participant is unsatisfied, the audiometrician is allowed to change the sound amplification gain between 90% and 110% or perform fine-tuning of specific frequencies depending on the participant’s feedback.

### Control group

The participants in the control group will receive standard care HA rehabilitation currently conducted at the KUH ENT clinic. Prescription HAs with custom earmoulds will be fitted using the manufacturers’ proprietary fitting algorithms and REM fitting will be performed only in complex cases, based on the clinical judgement of the audiometrician. In the control group, HA fittings are performed by audiometricians following routine clinical practice and who have not received specific training in the HAHA study procedures. Three months after the HA fitting, participants will be contacted via telephone and asked about the status of the rehabilitation. Additional monitoring visits may be offered based on participant feedback. Individual HA settings will be decided by the audiometrician based on clinical judgement and without predefined sound amplification gain levels. Further adjustments will be made only on a participant’s request. Due to the possible variability of the fitting procedures, the key rehabilitation process variables will be documented (eg, fitting verification method, number of follow-ups, types of adjustments and HA usage data) and reported descriptively.

### Statistics

Power calculations were based on the primary comparison, that is, the 12-month SPIN change measured using FMST and DIN in the standard care (control) versus DDHR (intervention) group. To our knowledge, no data were available on the effects of HA rehabilitation on performance in the Finnish-language SPIN tests. We assumed a 12-month mean (SD) SPIN change of −0.9 (1.2) dB in the control group, based on preliminary data from the KUH ENT clinic.^34^ We also assumed a 12-month mean (SD) SPIN change of −2 (2.5) dB in the intervention group, based on published data on HA acclimatisation in older adults under conditions comparable to the HAHA intervention (10). To show a between-group difference with 90% power and at a 2.5% significance level (Bonferroni correction for two primary outcomes), with a 10% drop-out rate during 12 months, a n=180 (90/group) sample size would be needed. The HAHA trial will recruit up to 200 participants to maintain power during the 12-month extended follow-up (assuming 20% drop-out for 2 years).

Mixed-effects regression models will be applied to analyse change in each of the primary outcomes (FMST and DIN tests) as a function of randomisation group, time (continuous in days from baseline) and group×time interaction. Short-term intervention effects will be analysed based on data from the baseline (without HA) and 12-month (with HA) visits. Long-term intervention effects will be analysed based on data from baseline (without HA), 12-month (with HA) and 24-month (with HA) visits. Non-linearity of change will be considered in the analysis of the long-term intervention effects.

Statistical significance will be evaluated using the Hochberg method to account for the two primary outcomes. Secondary outcomes will also be analysed using the appropriate generalised mixed-effects regression model, depending on the distribution of the outcome.

Analyses of auditory outcome measures will include both short- and long-term intervention effects as follows:

- Change in HERE, SSQ, listening effort questionnaire, response time and THI will be analysed as a function of randomisation group, time (continuous in days from baseline) and group×time interaction. Data from baseline and 12-month visits will be used to analyse short-term intervention effects, and data from baseline, 12- and 24-month visits will be used for longer-term effects.
- Change in HA usage will be analysed as a function of randomisation group, time (continuous in days from primary HA fitting) and group×time interaction. Data from the 3- and 12 month visits will be used to analyse short-term intervention effects, and data from the 3-, 12- and 24-month visits will be used for longer-term effects.

Analyses of cognitive outcomes, where a longer time is needed to observe significant change compared with auditory outcomes, will focus mainly on the longer-term effects, analysed based on data from baseline and 12- and 24-month visits.

Analyses of quality of life and other psychosocial outcome measures will include both short-term (baseline and 12months) and longer-term (baseline, 12- and 24 months) intervention effects. Additional information on sensitivity analyses, accounting for missing or spurious data, and reporting dropouts is presented in online supplemental material S5.

### Ethics and dissemination

#### Ethics approval and consent to participate

This study is conducted in accordance with the Declaration of Helsinki and the International Conference on Harmonisation for Good Clinical Practice (ICH GCP E6). The study protocol and relevant documents were approved by the Regional Medical Research Ethics Committee of the well-being Services County of North Savo (approval no. 697/2023). Informed consent is obtained from each participant before initiating the study-specific procedure. This trial has been registered at ClinicalTrials.gov (NCT06495268, first release 2 July 2024). The version number of the protocol document at the time of submission of this article was 2.1 (date: 11 November 2024).

The Trial Steering and Management Group (TSMG), comprising the co-principal investigators, senior investigators and relevant study staff, will oversee trial conduct and progress and meet at least monthly throughout the study. An independent Data and Safety Monitoring Board of 3–4 experts in clinical trials, research and statistics will review safety data and advise the TSMG. The co-PIs will oversee data quality and integrity, and the trial site (KUH) will be periodically monitored by representatives of the University of Eastern Finland Brain Research Unit.

Trained HAHA personnel will enter case report form data into the database. All entries will be approved by the primary investigator or designee, and all post-submission changes will be electronically audit-trailed and dated.

#### Availability of data and materials

The full protocol will be published with the main trial results. Trial data and samples will remain confidential until analysis and review are complete and may not be published without TSMG approval. Data and samples will be available for collaborative research after publication of the main results on TSMG-approved application. Findings will be disseminated through peer-reviewed publications, conference abstracts and other standard channels.

## Discussion

The primary scope of the HAHA study, as a proof-of-concept trial, is to test a novel precision medicine model for hearing rehabilitation by approaching hearing loss as a broader degenerative entity with multifaceted manifestations that are currently unaddressed in clinical practice. Our goal is to determine whether a structured, data-driven rehabilitation approach that optimises functional hearing performance leads to measurable differences in communication ability and whether these improvements are associated with neural or cognitive correlates assessed through secondary outcomes (eg, imaging and electrophysiological measures). Additionally, a key objective is methodological: to identify sensitive and feasible outcome measures for assessing long-term HA benefits that could be used in a full-scale RCT and for effective hearing rehabilitation in the future.

Despite the negative consequences of hearing loss and the benefits of HAs, their uptake remains relatively low, even in countries where they are provided free of charge.^35 36^ Additionally, a significant proportion of those who have prescribed HAs do not use them, with no or irregular use up to 80%.^37^ There is a lack of consensus on which outcomes are relevant for the reliable quantification of HA benefits or which are important for maintaining brain health.^38^ Cochrane reviews on hearing rehabilitation^6 39^ recommend that future research address evidence gaps by focusing on long-term outcomes (≥1 year), developing standardised outcome measures and designing studies that capture benefits beyond HA provision. The HAHA trial aims to address these recommendations by identifying and providing currently missing relevant outcomes for an accurate assessment of the effectiveness of hearing rehabilitation. Additionally, the trial seeks to establish an evidence-based approach to examine hearing loss as a broader degenerative condition, integrating aspects beyond just providing HAs. These aspects include optimised hearing rehabilitation, the association between cognitive decline and hearing loss, the impact of DSI on hearing rehabilitation outcomes and the effect of hearing loss on quality of life and other psychosocial outcomes. Eventually, the results of this study may inform future best practice guidelines for hearing rehabilitation.

More specifically, this trial aims to provide new evidence on auditory processing and neural mechanisms of hearing rehabilitation and its impact on cognition, as well as identify novel, accessible and non-invasive biomarkers predicting hearing rehabilitation benefits on cognitive decline (eg, EEG-derived CAEPs as predictors of hearing rehabilitation benefit; SPIN and retinal imaging biomarkers for cognitive decline).

The HAHA trial is based on a novel pragmatic approach that is fully embedded in routine specialised care. Hearing rehabilitation in Finland is centralised to the specialised hearing centres of district/university hospitals, where patients with hearing loss are referred from primary care. Specialised hearing centres follow a standard clinical protocol for hearing loss assessment and rehabilitation, and the costs of HAs are reimbursed by the well-being Services counties. At present, the standard clinical protocol in Finland includes only the fitting of HAs at the audiometrician’s appointment, and further appointments are arranged based only on patient feedback. The HA fitting method used is generally the initial fit, regardless of the recommendations to use REM-based fitting. Although the DDHR approach in this trial is designed to optimise functional hearing outcomes, its structured procedures may require additional clinical resources that are not necessarily available in routine audiology practice. Therefore, assessing feasibility and identifying scalable implementation strategies are important secondary objectives of this proof-of-concept study.

In the HAHA study, all trial participants will be fitted according to the standard care protocol for the control group and using the novel DDHR programme for the intervention group. The main difference between these rehabilitation groups is that the outcomes of the HA rehabilitation are verified for the intervention group, while the control group only receives the HAs, and the monitoring of the outcomes is done only via a phone call. The HAs provided for the study groups are the same as in the Finnish standard clinical protocol, but the HA fitting method used for the intervention group is REM-based fitting. This unique rehabilitation approach of the intervention group, with proper fitting and outcome verification, will be crucial to optimise the current hearing loss clinical pathway and treatment process.

Recruitment of clinical referral patients will provide genuine, real-world data on patients experiencing mild to moderately severe hearing loss,^1^ allowing the probability of bias in participant selection to be low. The study setting (RCT) and its notably large size (up to 200) are the strengths of this trial, as no previous RCTs of this kind have been conducted before.^6^ The primary limitations of the study are related to the pragmatic design of the trial and its logistical requirements. In addition, the study sample is restricted to individuals who are able to access services at KUH, which may limit the generalisability of the results. In particular, because the trial is embedded in a part of real clinical work, masking will inevitably be affected by this. The variability in the control arm is acknowledged; however, this reflects usual clinical practice and is essential for assessing the added value of a structured data-driven rehabilitation approach under real-world conditions. However, to mitigate variability, systematic tracking and reporting of key variables in the rehabilitation process of both study arms will be conducted. Another limitation is the relatively short follow-up time (short term, 12 months; longer term, 24 months; extended follow-up) for a reliable assessment of cognitive change. However, we plan to conduct a longer follow-up period (10 years) after the intervention.

## Supplementary material

10.1136/bmjopen-2026-122681 online supplemental file 1

10.1136/bmjopen-2026-122681 online supplemental file 2

10.1136/bmjopen-2026-122681 online supplemental file 3

10.1136/bmjopen-2026-122681 online supplemental file 4

10.1136/bmjopen-2026-122681 online supplemental file 5
