# Supplementary material for: Proof-of-concept randomised controlled trial of data-driven hearing rehabilitation versus standard care in older adults with hearing loss: the healthy hearing for healthy ageing protocol
Source: BMJ Open. 2026 Jul 21;16(7):e122681. doi: 10.1136/bmjopen-2026-122681 (PMC13404848; doi:10.1136/bmjopen-2026-122681)
Supplement: online supplemental file 2 [file bmjopen-16-7-s002.docx]

**Supplementary material S2. The consent form**

CONSENT TO PARTICIPATE IN RESEARCH

The effect of a new hearing rehabilitation method on functional hearing and memory
in patients undergoing hearing rehabilitation assessment.

***HAHA - Healthy hearing for Healthy Ageing***

North Savo Wellbeing Services County
Kuopio University Hospital
Department of Otolaryngology

University of Eastern Finland
Brain Research Unit

I, _______________________________________, have been asked to participate in the above-mentioned scientific research, **the purpose of which is to examine the impact of a new hearing rehabilitation method on patients’ auditory performance and the connection between hearing aid rehabilitation and the maintenance of cognitive functioning.**

I have read and understood the written research information provided to me. I have received sufficient
information about the research and the associated data collection, processing, and disclosure. The content of the information has also been explained to me orally, I have had the opportunity to ask questions, and I have received sufficient answers to all my questions regarding the research.

Information provided by: ________________________ __ / __/ 20 ___

I have had enough time to consider my participation in the study. I have received sufficient information
about my rights, the purpose of the study, its implementation, and the potential benefits and risks.
I have not been pressured or lured to participate in the study. I understand that my participation is voluntary. I know that my data will be treated confidentially.

**I give my consent that:**

− the information I provide on research forms, the data recorded in the patient information system,
results from hearing and vision tests, EEG measurement results, imaging studies, and blood samples
collected from me may be used for the scientific research described in the research information form.

− information from my medical records may be collected during the study. This data will be used not only for the research but also to ensure that any changes in my health during the study do not affect my participation.

− the data and samples collected during the study may be used in cooperation with the research partners mentioned in the information form for research and product development. Information provided to these partners will be anonymized so that participants cannot be identified.

I am aware that if I withdraw from the study, data collected from me up to that point may still be used
as part of the research material.

I am also aware that I can withdraw my consent to the study at any time without giving a reason.
In such cases, the data already collected will not be used for research purposes unless it has already been analyzed.

Furthermore, the data and samples collected from me in this study may be reused in future additional studies related to hearing, eye, and memory disorders. My data may also be shared with the partners mentioned in the information form and used jointly for research and product development.

**1) I consent to the reuse of the data collected during the HAHA project for future additional studies related to hearing, eye, and memory disorders. I also consent to my data being shared with the partners mentioned in the information form and used for joint research and product development.**

|  | Yes |  | No |
| --- | --- | --- | --- |

**2) I may be contacted in connection with potential additional studies.**

|  | Yes |  | No |
| --- | --- | --- | --- |

**3) I consent to the following optional examinations during the HAHA project:**

**Head MRI scan**

|  | Yes |  | No |
| --- | --- | --- | --- |

**With my signature, I confirm my participation in this study and voluntarily agree to be a research subject.**

| Paticipant’s name |  | Place and date | |  | Signature |
| --- | --- | --- | --- | --- | --- |
| Date of birth |  | Address |  |  |  |

**Consent received**

| Receiver of consent |  | Place and date |  | Signature |
| --- | --- | --- | --- | --- |

The original signed participant consent and a copy of the research information sheet will remain archived with the responsible researcher. The research information sheet and a copy of the signed consent form will be given to the participant.
