# Supplementary material for: Proof-of-concept randomised controlled trial of data-driven hearing rehabilitation versus standard care in older adults with hearing loss: the healthy hearing for healthy ageing protocol
Source: BMJ Open. 2026 Jul 21;16(7):e122681. doi: 10.1136/bmjopen-2026-122681 (PMC13404848; doi:10.1136/bmjopen-2026-122681)
Supplement: online supplemental file 3 [file bmjopen-16-7-s003.docx]

**Supplementary material. S3: Summary of the HAHA assessments schedule and data collection**

|  | POPULATION | CONDUCTED BY | SCREENING | BASELINE | Primary HA fitting (M0) | M3 | M6 | M12 | M24 |
| --- | --- | --- | --- | --- | --- | --- | --- | --- | --- |
| VISIT WINDOW  (from primary HA fitting, as per standard care schedule) | NA | NA | Pragmatic approach, standard care wait-time 3-6 months from first ENT clinic visit (HAHA screening) to primary HA fitting. Randomisation after baseline, at least 1-2 months before HA fitting as per standard care scheduling system, to assign ENT clinic staff trained as HAHA hearing rehabilitators to the intervention group. | | | ±1 month | ±1 month | ±1 month | ±1 month |
| ASSESSMENTS / PROCEDURES | | | | | | | | | |
| Consent | All | ENT assessor | X | X | X | X | X | X | X |
| ELIGIBILITY | | | | | | | | | |
| Inclusion criteria | | | | | | | | | |
| Age | All | ENT assessor | X |  |  |  |  |  |  |
| Hearing loss level (1) | All | ENT assessor | X |  |  |  |  |  |  |
| Community-dwelling | All | ENT assessor | X | X |  |  |  |  |  |
| Proficiency in Finnish language | All | ENT assessor | X |  |  |  |  |  |  |
| First-time HA user | All | ENT assessor | X |  |  |  |  |  |  |
| Exclusion criteria | | | | | | | | | |
| Conductive hearing loss | All | ENT assessor | X |  |  |  |  |  |  |
| Difference between the hearing levels of the ears | All | ENT assessor | X |  |  |  |  |  |  |
| HA contraindication | All | ENT assessor | X |  |  |  |  |  |  |
| Dementia status (medical records & self-reported) | All | ENT assessor | X | X |  |  |  |  |  |
| Other relevant health-related conditions | All | ENT assessor | X | X |  |  |  |  |  |
| GENERAL / COVARIATES | | | | | | | | | |
| Sociodemographics & other data | | | | | | | | | |
| Date of birth (medical records) | All | ENT assessor | X |  |  |  |  |  |  |
| Sex (medical records) | All | ENT assessor | X |  |  |  |  |  |  |
| Ethnicity | All | Self-reported |  | X |  |  |  |  |  |
| Education | All | Self-reported |  | X |  |  |  |  |  |
| Marital status | All | Self-reported |  | X |  |  |  |  |  |
| Yearly income | All | Self-reported |  | X |  |  |  |  |  |
| Occupation | All | BRU assessor |  | X |  |  |  |  |  |
| Work history including noise exposure | All | BRU assessor |  | X |  |  |  |  |  |
| Other risk factors relevant for dementia risk scores | All | Self-reported |  | X |  |  |  | X | X |
| Subjective memory questionnaire | All | Self-reported |  | X |  |  |  | X | X |
| BNSQ | All | Self-reported |  | X |  |  |  | X | X |
| Other lifestyle-related risk factors relevant for dementia risk scores | All | Self-reported |  | X |  |  |  | X | X |
| Pure-tone audiometry | All | ENT assessor | X (no HA) |  |  |  |  | X (no HA) | X (no HA) |
| Medical history | | | | | | | | | |
| History of cardiovascular diseases, diabetes etc | All | Self-reported |  | X |  |  |  | X | X |
| Current medication | All | Self-reported / BRU assessor | X | X |  |  |  | X | X |
| Family history of hearing loss, dementia, and other conditions increasing dementia risk (e.g. cardiovascular diseases, diabetes). | All | Self-reported |  | X |  |  |  |  | X |
| Incident dementia / MCI diagnosis | All | Self-reported |  | X |  |  |  | X | X |
|  |  | Medical records (ENT assessor) |  |  |  |  |  | X | X |
| Anthropometrics | | | | | | | | | |
| BMI | All | BRU assessor |  | X |  |  |  | X | X |
| Blood pressure | All | BRU assessor |  | X |  |  |  | X | X |
| Waist / hip ratio | All | BRU assessor |  | X |  |  |  | X | X |
| Heart rate | All | BRU assessor |  | X |  |  |  | X | X |
| PRIMARY OUTCOMES | | | | | | | | | |
| DIN | All | Self-administered |  | X (no HA) |  |  |  | X (±HA) | X (±HA) |
| FMST | All | ENT assessor |  | X (no HA) |  |  |  | X (±HA) | X (±HA) |
| SECONDARY OUTCOMES | | | | | | | | | |
| Auditory measures | | | | | | | | | |
| HERE | All | Self-administered |  | X |  |  |  | X | X |
| SSQ | All | Self-administered |  | X |  |  |  | X | X |
| HA usage | All | ENT assessor (HA log) |  |  |  |  |  | X | X |
|  | All | Self-reported |  |  |  | X |  | X | X |
| Listening effort questionnaire | All | Self-administered |  | X (no HA) |  |  |  | X (+HA) | X (+HA) |
| Response time | All | Automatically recorded and calculated |  | X |  |  |  | X | X |
| Tinnitus Handicap Inventory | All | Self-reported |  | X |  |  |  | X | X |
| Cognitive measures | | | | | | | | | |
| CERAD-nb | All | BRU assessor |  | X (no HA) |  |  |  | X (with HA) | X (with HA) |
| CDR-SoB | All | BRU assessor |  | X (no HA) |  |  |  | X (with HA) | X (with HA) |
| Quality of life and other psycho-social measures | | | | | | | | | |
| 15D | All | Self-reported |  | X |  |  |  | X | X |
| EQ5-D-5L |  | Self-reported |  | X |  |  |  | X | X |
| Beck Depression Inventory | All | Self-reported |  | X |  |  |  | X | X |
| * EXPLORATORY / OPTIONAL ASSESSMENTS | | | | | | | | | |
| Exploratory outcomes | | | | | | | | | |
| EEG-derived CAEPs | All | Clinical neurophysiology assessor |  | X |  |  |  | X | X |
| Structural MRI | All (volunteer) | Clinical radiology assessor |  | X |  |  |  |  | X |
| Measures of physically, socially, cognitively active lifestyle | All | Self-reported |  | X |  |  |  | X | X |
| Vision-related measures | | | | | | | | | |
| Best-corrected visual acuity | All | Ophthalmology assessor |  | X |  |  |  |  | X |
| Slit-lamp examination | All | Ophthalmology assessor |  | X |  |  |  |  | X |
| Retinal imaging | All | Ophthalmology assessor |  | X |  |  |  |  | X |
| Eye geometrics and biometric values | All | Ophthalmology assessor |  | X |  |  |  |  | X |
| Blood samples | | | | | | | | | |
| Frozen and stored for future assessment of hearing loss or dementia-related markers (e.g. genetic, amyloid, tau, neurodegeneration) | All | BRU assessor |  | X |  |  |  |  | X |
| INTERVENTION-RELATED ACTIVITIES | | | | | | | | | |
| HA fitting | Active | ENT assessor (HAHA trained) |  |  | X^a^ | Visits M3, M6, M12 and M24: HA re-fittings performed as needed based on participant feedback, DIN test results and/or clinical judgement on each visit. | | | |
|  | Control | ENT assessor (standard care) |  |  | X^b^ | Check via phone call | No routine follow-up visits; HA re-fittings arranged upon participant request. | | |

* Exploratory/optional assessments may deviate from the specified visit windows depending on EEG- and imaging-related site logistics, and to reduce participant burden as needed. Brain MRI scans should be conducted after EEG-derived CAEPs in connection to the baseline visit.

^a^With the Real-Ear Measurements

^b^With Initial-Fit

1. Haile LM, Kamenov K, Briant PS, Orji AU, Steinmetz JD, Abdoli A, et al. Hearing loss prevalence and years lived with disability, 1990-2019: Findings from the Global Burden of Disease Study 2019. The Lancet. 2021 Mar 13;397(10278):996–1009. doi:10.1016/S0140-6736(21)00516-X PubMed PMID: 33714390.
