# Supplementary material for: Proof-of-concept randomised controlled trial of data-driven hearing rehabilitation versus standard care in older adults with hearing loss: the healthy hearing for healthy ageing protocol
Source: BMJ Open. 2026 Jul 21;16(7):e122681. doi: 10.1136/bmjopen-2026-122681 (PMC13404848; doi:10.1136/bmjopen-2026-122681)
Supplement: online supplemental file 4 [file bmjopen-16-7-s004.docx]

**Supplementary material S4. The measurement tools for secondary outcomes**

| The name of the test | Explanation | Example |
| --- | --- | --- |
| Hearing in real-life environment (HERE) (22) | -self-reporting hearing questionnaire validated in Finnish language  -includes 15 items with a numeric rating scale from 0 to 10 for each item and allows the assessment of hearing with and without hearing aids | *“I will have to make an effort when I listen to something or someone.”* |
| Speech, Spatial and Quality questionnaires (SSQ) (23) | -self-reporting hearing questionnaire that comprises 49 questions divided into three subdomains: Speech Perception (SP), Spatial Hearing (SH) and other qualities of Hearing (SQ)  -answers are provided on an 11-point Likert scale, ranging from 0 to 10 | *“You are sitting around a table in a quiet room with four people. You can see each of them around you. Can you follow the conversation?”* |
| Listening effort questionnaire (24) | -self-reporting questionnaire for listening tasks of varying difficulties (i.e., different signal-to-noise ratios) on a 10-point VAS scale | *“On the scale from 0 to 10, describe how much effort you have to put into listening situations.”* |
| Tinnitus Handicap Inventory (THI) (25) | -25-item self-report questionnaire for tinnitus that has Functional, Emotional and Catastrophic subscales  -answers are provided with three words (yes, sometimes, no)  -scoring: 4 for a ‘yes’, 2 for ‘sometimes’ and 0 for ‘no’ | *“Do you have difficulties with concentrating because of tinnitus?”* |
| Consortium to Establish a Registry for Alzheimer´s Disease (CERAD-nb, global and domain scores) (26) | -for assessment of memory disorders  -the test battery includes:  1) CERAD modified 15-item Boston Naming Test; a wide-range naming vocabulary test consisting of 15 pictures ordered from easiest to most difficult. Pictures are presented allowing subject up to 20 seconds for response.  2) CERAD category fluency; measures the ability to recollect as many words as possible that belong to a certain category. The score is the number of different animals named in 60 seconds.  3) the Mini-Mental State Examination (MMSE); is a 30-item mental status questionnaire that assesses a participant’s mental status (orientation, memory, attention, language, visual-spatial abilities, and calculation). A total MMSE score is calculated by summing of all correct items out of a possible 30 points.  4) CERAD, 10-Word Recall Task (10-word learning, recall and recognition); the participant is given three trials to learn a list of high-frequency, high-imagery nouns. The 10 words are printed in block letters on white cards and the participant is asked to read every word aloud. After each rehearsal the participant is asked to recall as many words as he /she can (learning). After a short delay (5-10 minutes) the participant is asked to again recall the words (delayed recall). In the last part of the participant is asked to recognise the formerly learned words from the separate word list (recognition).  5) CERAD Constructional Praxis; this is a measure for visual perception and constructional praxis. The task is administered by asking the participant to copy four drawings that are presented one by one. There is no time limit and maximum total score for the task is 11 points.  6) CERAD Constructional Praxis recall; this is a measure for visual memory and memory based constructional praxis. After a 5-to-10-minute delay the participant is asked to draw every figure that he/she is able to remember from CERAD Constructional Praxis task. There is no time limit and total score for the task is 11 points.  7) CERAD Clock Drawing Test (CDT): the participant is instructed to draw a clock by first drawing a circle, then adding numbers, and then setting the time at 11:10. There is no time limit and the maximum total score for the test is 6 points. | *-*  *-*  *“Can you tell me, what year is it now?”*  *-*  *-*  *-*  *-* |
| Clinical Dementia Rating Sum of Boxes (CDR-SoB) (27) | -for assessment of memory disorders  -the assessor will interview the participant on six domains: memory, orientation, judgment and problem solving, community affairs, home and hobbies, and personal care  -each domain is rated on level of impairment: 0 (none), 0.5 (questionable), 1 (mild dementia), 2 (moderate dementia) or 3 (severe dementia)  -the total Sum of Boxes ranges from 0 (no impairment) to 18 (severe impairment on all domains) | *“Have you had any problems due to memory or reasoning difficulties?”* |
| 15D-questionnaire (28) | -self-administered questionnaire of health-related quality of life developed in Finland  -includes 15 questions providing a numerical score for patient-perceived health status  -can be used both as a profile and single index score measure  -commonly used in Finland to assess quality of life in standard care | *”I am able to walk normally (without difficulty) indoors, outdoors, and on stairs.”*  *”I am completely immobile and bedridden.”* |
| EQ5-D-5L questionnaire (29) | -concise, generic measure of self-reported health which is accompanied by weights reflecting the relative importance to people of different types of health problems | *“I have no difficulties washing myself or dressing.”*  *“I am unable to wash or dress myself.”* |
| Beck Depression Inventory (BDI) (30) | - questionnaire for the assessment of depression in clinical practice, including in older adults  -self-administered questionnaire to quantify psychological and physical symptoms of depression  -includes 21 items, and participants are asked to rate each item using a 4-point Likert scale | *“I do not feel sad.”  “I am so sad or unhappy that I cannot bear it.”* |
