# Supplementary material for: Proof-of-concept randomised controlled trial of data-driven hearing rehabilitation versus standard care in older adults with hearing loss: the healthy hearing for healthy ageing protocol
Source: BMJ Open. 2026 Jul 21;16(7):e122681. doi: 10.1136/bmjopen-2026-122681 (PMC13404848; doi:10.1136/bmjopen-2026-122681)
Supplement: online supplemental file 5 [file bmjopen-16-7-s005.docx]

**Supplementary material S5.**

**Sensitivity analyses, procedures for missing data and reporting dropouts**

#### **1) Sensitivity and subgroup analyses**

#### A secondary analysis of the primary outcomes will be conducted for the per-protocol (PP) population, defined as a subset of the intention-to-treat (ITT) population without major protocol deviations, as follows:

- Violations in inclusion and exclusion criteria at enrolment

- Complete discontinuation of hearing aid (HA) use due to concomitant health conditions (e.g., repeated external ear canal infection or serious skin condition; participant unable to use HA due to incident cognitive or other physical problems)

- Participants identified during study procedures to not have any hearing benefits from the HA, for whom the possibility the cochlear implant surgery is considered as per standard care procedure at the Kuopio University Hospital Hearing Center.

All major protocol deviations will be identified in a blinded fashion prior to database lock.

Sensitivity analyses will be done with a modified intention-to-treat population (mITT, all randomly assigned participants with at least one post-baseline observation), all randomly assigned participants with a multiple imputation method, and all randomly assigned participants who completed all auditory assessments. A similar approach will be used for cognitive outcomes; in addition, binary logistic regression analyses will be conducted with outcome defined as cognitive decline versus improvement or no change between assessments at baseline and 24 months.

Subgroup analyses will be conducted on potential intervention effect modificators: socio-demographics (e.g., age, sex), socioeconomic status (e.g., income, education), HA compliance, hearing loss severity, baseline cognition, risk of dementia (e.g. based on risk scores).

**2) Procedures to account for missing or spurious data**

Outcome assessments will be conducted by trained HAHA team members with relevant experience (e.g. audiology, dementia-related cognitive testing). Self-administered assessments (the Digits-in-Noise-test) will be done on site at the Kuopio University Hospital Hearing Center, with a HAHA team member available for technical support to ensure completion. To prevent missing of self-reported data, questionnaires will be checked and discussed during assessment visits, participants will have the possibility to ask questions or explanations about the questionnaires or receive help to complete them.

Individual reason for missing data (e.g., assessment not conducted, refused to answer, unable to perform the task) will be reported in the relevant case report forms and will be assigned a unique numerical code in the database.

Missing values in outcome parameters will be considered as Missing At Random (MAR) in the analysis. Mixed models are able to handle unbalanced and incomplete repeated measures data, implying the more common assumption of MAR, rather than the strict and less likely Missing Completely At Random (MCAR) assumption on the response parameter.

For missing on covariates, missingness mechanism of MCAR will be assumed, therefore a single imputation of the overall mean of the baseline value will be incorporated in the primary analysis. In general, if there are more than 30% missing cases, multiple imputation will be considered as a sensitivity analysis.

**3) Reporting dropouts**

Information will be provided on number of dropouts in each trial group, timing of dropouts, and reasons for dropout. Comparisons between individuals who drop out and those who do not will be conducted, e.g., via T-tests, cross tabulations, or logistic regression. To reduce missing data, dropouts will also be invited to the 24-month outcome assessment visit.
