# Supplementary material for: Proof-of-concept randomised controlled trial of data-driven hearing rehabilitation versus standard care in older adults with hearing loss: the healthy hearing for healthy ageing protocol
Source: BMJ Open. 2026 Jul 21;16(7):e122681. doi: 10.1136/bmjopen-2026-122681 (PMC13404848; doi:10.1136/bmjopen-2026-122681)
Supplement: Supplementary file 6 [file bmjopen-2026-122681.reviewer_comments.pdf]

PEER REVIEW HISTORY

BMJ Open publishes all reviews undertaken for accepted manuscripts. Reviewers are asked to complete a checklist review form and are provided with free text boxes to elaborate on their assessment. These free text comments are reproduced below.

ARTICLE DETAILS

Title (Provisional)

A proof-of-concept randomised controlled trial of data-driven hearing rehabilitation versus standard care in older adults with hearing loss: the Healthy Hearing for Healthy Ageing (HAHA) protocol

Authors

Ihalainen, Laura; Barbera, Mariagnese; Törmäkangas, Timo; Hyvärinen, Petteri; Willberg, Tytti; Linder, Pia; Solomon, Alina; Dietz, Aarno

---

VERSION 1 - REVIEW

**Reviewer**	**1**
**Name**	**Robler, Samantha**
**Affiliation**	**University of Arkansas for Medical Sciences**
**Date**	**18-Jun-2026**
**COI**	**None**

A proof-of-concept randomised controlled trial of datadriven hearing rehabilitation versus standard
care in older adults with hearing loss: the Healthy Hearing for Healthy Ageing (HAHA) protocol

This is a well-presented protocol for a randomized controlled trial evaluating the effectiveness of data-driven hearing rehabilitation (DDHR) compared with standard care on functional hearing benefit and cognitive decline in older adults with hearing loss. Recruitment began in October 2024 and will continue for two years, allowing for 12- and 24-month follow-up assessments.

The protocol is comprehensive and generally well described. The primary outcome, speech-in-noise performance, is clearly defined and will be assessed using two measures (FMST and DIN). Secondary outcomes focus on patient-reported measures, including hearing-related quality of life, listening effort, tinnitus, depression, cognition, and general health. Exploratory outcomes examine cortical and neural changes, as well as the impact of DSI on cognition.

In terms of presentation and clarity, I have a few minor suggestions. Most notably, the authors should more clearly define what constitutes "standard care" (e.g., page 8, line 10, and page 9 following the introduction of DDHR). A more detailed description of standard care would help readers better understand the basis for the authors' assertion that DDHR may provide a more

effective approach to hearing rehabilitation.

Similarly, although the differences in appointment structure and follow-up procedures between the two study arms are well described, the use of real-ear measurements (REM) is less clear. The authors should clarify that while REM is considered a best-practice recommendation, it is not routinely implemented in this clinical setting and is generally reserved for more complex cases based on clinical judgment. Include references where possible. This context is important for understanding the distinction between the intervention and control conditions.

Finally, it would be helpful to provide additional detail regarding who delivers the interventions and who conducts outcome assessments in this pragmatic clinical setting. Specifically, further description of the respective roles of the audiometrician and audiologist, as well as the personnel responsible for outcome assessments, would improve transparency regarding study implementation and potential sources of bias, particularly for the control arm.

Overarching Comment

Because the intervention is intentionally embedded within a real-world clinical environment, additional detail regarding usual care practices and implementation workflows would substantially strengthen the protocol and improve reproducibility and interpretation of future findings. In particular, a clearer description of standard care, including the routine use (or non-use) of real-ear measurements, the personnel responsible for intervention delivery and outcome assessments, and the clinical pathways followed in each study arm, would help readers better understand the pragmatic context in which the intervention is being evaluated and the factors that may influence implementation and effectiveness.

Line-by-Line

Comments Abstract

- Page 3, line 42: Consider including DSI among the exploratory outcomes.

Introduction

- There are a few awkward phrases that could be revised for readability. For example:
    - Page 5, line 12: Consider removing "level of."
    - Page 6, line 24: Consider revising to, "Older adults, in particular, …"
- Minor formatting issues are present and can likely be addressed during proofing (e.g., page 7, line 24, punctuation associated with the in-text citation).

Methods

- Page 9, line 17: There has been a gradual shift away from the term "blind" in favor of "masked." The authors may wish to consider adopting this terminology, particularly given the emphasis on DSI within the study. See:

  https://pmc.ncbi.nlm.nih.gov/articles/PMC1851996/

- Page 10, line 5: The protocol excludes individuals with severe vision loss that precludes participation yet includes both a vision examination and exploratory

evaluation of DSI. Additional clarification regarding how vision-related eligibility is determined would be helpful.

- Page 10, line 12: Consider changing to present tense, as the study is currently enrolling participants.
- Page 10, lines 21–22: The assumptions underlying the power calculation are difficult to follow. Consider revising this sentence and the following one for clarity.

Discussion

- Page 20, lines 20–21: This section helps clarify the rationale for comparing standard care with DDHR; however, these concepts are not sufficiently introduced in the background or methods. The manuscript would benefit from earlier discussion of both REM as a best-practice recommendation and the clinic's reliance on the initial hearing aid fitting without routine REM verification.
- Page 20, lines 21–33: The authors make an important point regarding feasibility and scalable implementation strategies. However, these appear to be characterized as important secondary objectives without being explicitly introduced in the background or methods sections. Consider incorporating these objectives earlier in the manuscript.
- Page 21, lines 8–23: I agree with the authors regarding the value of conducting a pragmatic, clinic-based trial. However, it is also worth acknowledging that the sample is limited to individuals who are able to access services at KUH, which may affect generalizability.

Conclusion

- I defer to the journal regarding inclusion of a conclusion section, as conclusions are not always included in protocol papers. If retained, the section is appropriate as written.

Tables

- Table 2: I was surprised by footnote *g*, which indicates that both groups receive REAR assessments at all time points. This appears inconsistent with Supplemental Table S3, which suggests that the control group only receives REAR assessments beyond the initial fitting when clinically indicated. Please clarify.

Supplemental Material

- I was unable to access Supplemental File S2 (Informed Consent).
- Supplemental Table S3: It is unclear why the M6 time point is blank for consent while all other time points are indicated. Please clarify.

VERSION 1 - AUTHOR RESPONSE

REVIEWER #1 COMMENTS:

A proof-of-concept randomised controlled trial of datadriven hearing rehabilitation versus standard care in older adults with hearing loss: the Healthy Hearing for Healthy Ageing (HAHA) protocol

This is a well-presented protocol for a randomized controlled trial evaluating the effectiveness of data-driven hearing rehabilitation (DDHR) compared with standard care on functional hearing benefit and cognitive decline in older adults with hearing loss. Recruitment began in October 2024 and will continuefor two years, allowing for 12- and 24-month follow-up assessments.

The protocol is comprehensive and generally well described. The primary outcome, speech-in-noise performance, is clearly defined and will be assessed using two measures (FMST and DIN). Secondary outcomes focus on patient-reported measures, including hearing-related quality of life, listening effort, tinnitus, depression, cognition, and general health. Exploratory outcomes examine cortical and neural changes, as well as the impact of DSI on cognition.

In terms of presentation and clarity, I have a few minor suggestions. Most notably, the authors should more clearly define what constitutes "standard care" (e.g., page 8, line 10, and page 9 following the introduction of DDHR). A more detailed description of standard care would help readers better understand the basis for the authors' assertion that DDHR may provide a more effective approach to hearing rehabilitation.

Response: Thank you for this helpful suggestion. We agree that a clearer description of standard care improves the readability of the manuscript and provides important context for understanding the rationale for comparing it with DDHR. We have therefore revised the relevant sections to provide a more detailed description of the Finnish standard hearing rehabilitation pathway, including the use of manufacturer-prescribed automatic fitting algorithms as well as the limited use of REM, and the routine follow-up practices. These revisions clarify the characteristics of standard care and better distinguish it from the DDHR programme. (page 8, lines 5-8; page 9, lines 5-17)

Similarly, although the differences in appointment structure and follow-up procedures between the two study arms are well described, the use of real-ear measurements (REM) is less clear. The authors should clarify that while REM is considered a best-practice recommendation, it is not routinely implemented in this clinical setting and is generally reserved for more complex cases based on clinical judgment. Include references where possible. This context is important for understanding the distinction between the intervention and control conditions.

Response: Thank you for this comment. The rationale for selecting REM as the preferred method for HA fitting in DDHR programme is based on the recommendations of the latest ISO standard (ISO 21388:2020), a recent review article on best practices for HA fitting, and our preliminary findings from the BREM study. We have now clarified this rationale and the

supporting evidence in the Methods section, as suggested. (page 14, lines 8-14)

Finally, it would be helpful to provide additional detail regarding who delivers the interventions and who conducts outcome assessments in this pragmatic clinical setting. Specifically, further description of the respective roles of the audiometrician and audiologist, as well as the personnel responsible for outcome assessments, would improve transparency regarding study implementation and potential sources of bias, particularly for the control arm.
Response: Thank you for this comment. In Finland, the term audiologist refers to an otolaryngologist (ENT physician) with specialized training in audiology. To avoid confusion for an international readership, we have removed this term from the manuscript. HA fittings in Finland are routinely performed by audiometricians, who are registered nurses with additional training in audiological procedures, including HA fitting and audiological assessments.
In the HAHA study, HA fittings in the control group are performed by audiometricians delivering routine clinical care, whereas fittings in the DDHR group are performed by audiometricians who have received specific training in the HAHA study procedures. All outcome assessments are conducted by study nurses specifically trained in the HAHA study procedures, who are masked to participants' group allocation. We have clarified these roles and procedures in the Methods section to improve transparency (page 11, lines 10-12; page 14, lines 14-15; page 15, lines 17-19).
Overarching Comment
Because the intervention is intentionally embedded within a real-world clinical environment, additional detail regarding usual care practices and implementation workflows would substantially strengthen the protocol and improve reproducibility and interpretation of future findings. In particular, a clearer description of standard care, including the routine use (or non-use) of real-ear measurements, the personnel responsible for intervention delivery and outcome assessments, and the clinical pathways followed in each study arm, would help readers better understand the pragmatic context in which the intervention is being evaluated and the factors that may influence implementation and effectiveness.

Line-by-Line Comments

Abstract

• Page 3, line 42: Consider including DSI among the exploratory outcomes.

Response: Vision-related measures are included among exploratory outcomes as requested. (Page 3, line 9)

Introduction
• There are a few awkward phrases that could be revised for readability. For example:
o Page 5, line 12: Consider removing "level of."

Response: The phrase is corrected as requested. (Page 5, line 5)

o Page 6, line 24: Consider revising to, "Older adults, in particular, …"

Response: The phrase is corrected as requested. (Page 6, line 14)

• Minor formatting issues are present and can likely be addressed during proofing (e.g., page 7, line 24, punctuation associated with the in-text citation).

Response: The punctuation is corrected as requested. (Page 7, line 10)

Methods

• Page 9, line 17: There has been a gradual shift away from the term "blind" in favor of "masked." The authors may wish to consider adopting this terminology, particularly given the emphasis on DSI within the study. See: https://pmc.ncbi.nlm.nih.gov/articles/PMC1851996/

Response: The wording ”blinded/blinding” has been replaced with the wording ”masked/masking” throughout the manuscript as requested.

• Page 10, line 5: The protocol excludes individuals with severe vision loss that precludes participation yet includes both a vision examination and exploratory evaluation of DSI. Additional clarification regarding how vision-related eligibility is determined would be helpful.

Response: We thank the reviewer for this helpful comment. As noted in Table 1, no predefined visual acuity threshold is used as an exclusion criterion. Instead, eligibility is determined based on the clinical judgment of the study nurse and/or physician, who assess whether a participant's visual impairment is severe enough to prevent participation in the study and reliable completion of the touchscreen-based Digits-in-Noise (DIN) test. To improve clarity, we have revised the exclusion criterion in Table 1 to explicitly state that no predefined visual acuity threshold is applied.

• Page 10, line 12: Consider changing to present tense, as the study is currently enrolling participants.

Response: Thank you for this comment. We were not entirely sure which specific sentence the comment referred to. However, if it concerns the sample size and power calculation section that is now on page 16, we have substantially revised this section and corrected the grammatical tenses.

• Page 10, lines 21–22: The assumptions underlying the power calculation are difficult to follow. Consider revising this sentence and the following one for clarity.

Response: Thank you for this helpful comment. We have revised the section describing the sample size calculation and moved it to the Statistics section. Specifically, we now state that the power calculation was based on the primary comparison of the between-group difference in the 12-month change in speech-in-noise (SPIN) performance, measured using the Finnish Matrix Sentence Test (FMST) and the Digits-In-Noise (DIN) test. We have also clarified the assumptions underlying the calculation, including the expected mean improvements in the DDHR and standard care groups, the significance level, statistical power, and the anticipated dropout rates. In addition, we now describe the evidence supporting these assumptions, including published data on HA acclimatization and a dataset originally collected for a master's thesis in medicine.

The sample size calculation was performed using Stata version 19 with the following command: power two means -0.9 -2, sd1(1.2) sd2(2.5) alpha(0.025), power(0.9). This produces a sample size of 162 (81 per group). To account for 10% dropout, N=162 / 0.9 =180 (i.e. 162 represents 90% of 180).

(page 16, lines 7-18)

Discussion

• Page 20, lines 20–21: This section helps clarify the rationale for comparing standard care with DDHR; however, these concepts are not sufficiently introduced in the background or methods. The manuscript would benefit from earlier discussion of both REM as a best-practice recommendation and the clinic's reliance on the initial hearing aid fitting without routine REM verification.

Response: Thank you for this comment. As mentioned already earlier, we have revised the relevant sections in the Background and Methods to provide a more detailed description of the Finnish standard hearing rehabilitation pathway, including the use of manufacturer-prescribed automatic fitting algorithms as well as the limited use of REM, and the routine follow-up practices. These revisions clarify the characteristics of standard care and better distinguish it from the DDHR intervention.

• Page 20, lines 21–33: The authors make an important point regarding feasibility and scalable implementation strategies. However, these appear to be characterized as important secondary objectives without being explicitly introduced in the background or methods sections. Consider incorporating these objectives earlier in the manuscript.

Response: Thank you for this important comment. We have revised the Background section to include a discussion of the importance of assessing the feasibility of the DDHR approach and its potential implementation in routine clinical practice. (page 8, lines 19-23)

• Page 21, lines 8–23: I agree with the authors regarding the value of conducting a pragmatic,

clinic-based trial. However, it is also worth acknowledging that the sample is limited to individuals who are able to access services at KUH, which may affect generalizability.

Response: We thank the reviewer for this valuable comment. We have revised the manuscript accordingly by adding this point to the discussion of the study limitations (page 22, lines 8-9).

Conclusion
• I defer to the journal regarding inclusion of a conclusion section, as conclusions are not always included in protocol papers. If retained, the section is appropriate as written.

Response: The Conclusion section is removed as requested.

Tables
• Table 2: I was surprised by footnote g, which indicates that both groups receive REAR assessments at all time points. This appears inconsistent with Supplemental Table S3, which suggests that the control group only receives REAR assessments beyond the initial fitting when clinically indicated. Please clarify.

Response: We thank the reviewer for pointing out this lack of clarity. In the DDHR group, HA fitting using REM is routinely performed during the initial HA fitting session. Subsequent re-fittings are carried out as needed based on DIN-test results, participant feedback, and/or clinical judgment. In the control group, the initial HA fitting is performed using the HA manufacturer's proprietary fitting algorithm. REM is used only in complex cases, based on the clinical judgment of the hearing care professional. We have clarified these procedures in both Table 2 and Supplementary Table S3.

Supplemental Material
• I was unable to access Supplemental File S2 (Informed Consent).

Response: We thank the reviewer for bringing this to our attention. We have re-uploaded Supplemental File S2 to ensure that it is accessible and can be opened correctly.

• Supplemental Table S3: It is unclear why the M6 time point is blank for consent while all other time points are indicated. Please clarify.
Response: We want to thank the reviewer for noticing this error. The consent is indicated on every visit and Supplemental Table 3 is now corrected with X-mark also on the time point M6.
